# The Capacity of the Fecal Microbiota From Malawian Infants to Ferment Resistant Starch

**DOI:** 10.3389/fmicb.2019.01459

**Published:** 2019-06-26

**Authors:** Yanan Wang, Elissa K. Mortimer, Kondwani G. H. Katundu, Noel Kalanga, Lex E. X. Leong, Geetha L. Gopalsamy, Claus T. Christophersen, Alyson C. Richard, Aravind Shivasami, Guy C. J. Abell, Graeme P. Young, Geraint B. Rogers

**Affiliations:** ^1^Infection and Immunity Theme, South Australian Health and Medical Research Institute, Adelaide, SA, Australia; ^2^SAHMRI Microbiome Research Laboratory, School of Medicine, Flinders University, Adelaide, SA, Australia; ^3^Flinders University Global GI Health Unit, College of Medicine and Public Health, Flinders University, Bedford Park, SA, Australia; ^4^Division of Physiology, Biomedical Sciences Department, College of Medicine, University of Malawi, Blantyre, Malawi; ^5^Department of Health Systems and Policy, School of Public Health, College of Medicine, University of Malawi, Blantyre, Malawi; ^6^School of Medical & Health Sciences, Edith Cowan University, Joondalup, WA, Australia; ^7^School of Molecular & Life Sciences, Curtin University, Perth, WA, Australia

**Keywords:** high-amylose maize starch, weaning, fermentation, short chain fatty acids, fecal microbiota

## Abstract

In Low and Middle-Income Countries (LMIC), weaning is associated with environmentally acquired and inflammation-associated enteric disorders. Dietary intake of high amylose maize starch (HAMS) can promote commensal fermentative bacteria and drive the production of short chain fatty acids (SCFAs). By stabilizing commensal gut microbiology, and stimulating the production of anti-inflammatory metabolites, HAMS supplementation might therefore influence enteric health. However, the extent to which the gut microbiota of LMIC infants are capable of fermenting HAMS is unclear. We assessed the capacity of the fecal microbiota from pre-weaning and weaning Malawian infants to ferment HAMS and produce SCFAs using an *in vitro* fermentation model. Fecal microbiota from both pre-weaning and weaning infants were able to ferment HAMS, as indicated by an increase in bacterial load and total SCFA concentration, and a reduction in pH. All of these changes were more substantial in the weaning group. Acetate production was observed with both pre-weaning and weaning groups, while propionate production was only observed in the weaning group. HAMS fermentation resulted in significant alterations to the fecal microbial community in the weaning group, with significant increases in levels of *Prevotella, Veillonella*, and *Collinsella* associated with propionate production. In conclusion, fecal microbiota from Malawian infants before and during weaning has the capacity to produce acetate through HAMS fermentation, with propionate biosynthetic capability appearing only at weaning. Our results suggest that HAMS supplementation might provide benefit to infants during weaning.

## Introduction

Infants in Low and Middle-Income Countries (LMIC) are at high risk of environmentally acquired and inflammation-associated enteric disorders, including Environmental Enteric Dysfunction (EED). EED is an environmentally acquired gut disorder characterized by global disturbance of intestinal structure and function (Owino et al., 2016) that results from the cumulative impact of enteric infections. The characteristics of EED include enteric and systemic inflammation (Syed et al., 2016; Harper et al., 2018) and the condition is strongly associated with undernutrition, reduced growth rate and cognitive defects in LMIC infants (Owino et al., 2016). Poor sanitation and the microbial contamination of food and water supplies in LMIC contribute to high rates of enteric infection and EED (Korpe and Petri, 2012; Ali et al., 2016).

While the pathogenesis of EED is complex, the condition is both preventable and reversible (Owino et al., 2016). In addition to limiting the exposure of infants to enteric pathogens, reducing inflammation and restoring commensal gut microbiology are believed to be potential therapeutic strategies (Syed et al., 2016). Prebiotics might therefore provide benefit, by supporting the growth of commensal microbes, and by promoting the production of beneficial microbial metabolites (Gibson et al., 2017), including short chain fatty acids (SCFAs). SCFAs (predominantly acetate, butyrate and propionate) reduce local and systemic inflammation (Vinolo et al., 2011), and butyrate contributes to the maintenance of gut barrier function (Peng et al., 2009; Canani et al., 2011).

The potential prebiotic properties of high amylose maize starch (HAMS), a type 2 dietary resistant starch (RS), have been demonstrated previously (Zaman and Sarbini, 2016). HAMS promotes the growth of important commensal bacteria, including members of the *Bifidobacterium* genus. HAMS also drives SCFA production (Morita et al., 1998; Le Leu et al., 2009), a function that can result in reduced inflammation and improved gut health, as demonstrated both rodent models (Le Leu et al., 2009) and healthy adults (van Munster et al., 1994).

The transition from exclusive breastfeeding to complementary food intake (weaning) presents heightened risks for EED due to for the increased exposure to enteric pathogens through faeco-oral contamination (Motarjemi et al., 1993; Crane et al., 2015). In Malawi, the prevalence of diarrhoeal diseases rises sharply from 13% before weaning age to 41% at 6–12 months (National Statistical Office and The DHS Program ICF, 2017). Enteric health during this period is therefore likely to be critical in EED development. However, weaning also represents a first opportunity for dietary interventions that stabilize commensal gut microbiology and reduce inflammation.

HAMS has Generally Recognized as Safe (GRAS) status. Additionally, it is inexpensive, and does not alter the taste or textural properties when added to food (Brown, 1996). Maize is a common food in Malawi, used widely as a weaning food (Kerr et al., 2007). This enables the ready addition of HAMS to typical weaning foods, without disrupting existing dietary practices. Because HAMS is highly resistant to human digestive enzymes, it passes into the large intestine to provide a potential fermentative substrate, allowing increased production of SCFAs. However, the few studies that have assessed the potential of HAMS supplementation to support gut health in LMIC children have produced inconsistent findings. A study in Indian children aged between 6 months to 3 years reported that the addition of HAMS to oral rehydration solution (ORS) significantly reduced the duration of diarrhea, compared to standard glucose ORS treatment alone (Raghupathy et al., 2006), suggesting that this approach can provide benefit. In contrast, the provision of doughnuts containing 8.5 g/d of HAMS to stunted Malawian children aged 3–5 years resulted in no improvement of gut inflammation, with increased levels of the gut inflammatory marker calprotectin, and decreased levels of acetate, compared to placebo (Ordiz et al., 2015).

Weaning of infants in Malawi typically starts at a younger age than the 6 months (Kerr et al., 2007) recommended by the World Health Organization [WHO], 2018. It is unclear whether the gut microbiota is sufficiently developed in infants of this age to ferment HAMS, and thereby provide benefit, given the typical absence or low abundance of bacterial clades able to ferment polysaccharides (Arrieta et al., 2014; Backhed et al., 2015). Therefore, before HAMS can be assessed as a potential strategy to prevent or treat EED, the capacity of the gut microbiota of infants in low-income country settings to ferment HAMS and produce SCFAs must be determined.

We report an investigation of the capacity of fecal microbiota from Malawian infants to utilize HAMS and produce SCFAs within an *in vitro* fermentation system, and describe the impact of this process on the composition of fecal microbiota. To assess the development of fermentative capacity during this transitionary period, and therefore whether HAMS might represent a potential first weaning food, our analysis included samples from both weaning and pre-weaning infants.

## Materials and Methods

### Study Population

The study was conducted according to the principles expressed in the Declaration of Helsinki, and all research procedures were approved by the Southern Adelaide Clinical Human Research Ethics Committee (SACHREC) for Flinders University (504.14-HREC/14/SAC/541) and the University of Malawi College of Medicine Research Ethics Committee (COMREC) (P.08/15/1781). In addition, a study approval letter was provided through the Neno District Health Office, authorizing the involvement of the Neno District Hospital. Written informed consent was obtained from all caregivers of the participating children.

Fecal samples were collected from infants attending the Neno District Hospital for routine child health checks between November 2015 and March 2016. Inclusion criteria for the participating infants were full-term pregnancy (≥37 weeks gestation) and vaginal birth. Infants who had antibiotic exposure or acute gastrointestinal illness within 2 weeks prior to sample collection, or had acute or major systemic disease, including HIV/AIDS, were excluded. Feeding practices reported by caregivers were recorded using a standardized template. Infants who were exclusively breast fed were classified as “pre-weaning.” Infants within 5 months of commencing complementary foods (partially breast fed) were classified as “weaning.”

### Sample Collection

Stool collection was performed using a disposable nappy with a plastic lining insert, which was fastened to the infant by study staff. Caregivers were asked to alert study staff when the infant had passed a stool. Nappies were removed immediately and a sterile spatula was used to transfer stool to 2 mL cryovials. Samples were immediately frozen in liquid nitrogen for shipment to the South Australian Health and Medical Research Institute, Adelaide, for analysis. Five samples were excluded due to insufficient volume for conducting the fermentation assay (<300 mg) and 34 samples were used for *in vitro* fermentation.

### *In vitro* Fermentation

To simulate gastric and small intestinal starch digestion in infants, HAMS (Hylon VII, Ingredion Incorporated) was pre-digested by an *in vitro* method adapted from a previous study (Woolnough et al., 2010; Wang et al., 2018). Full details of HAMS pre-digestion and preparation procedures are included as Supplementary Methods. The pre-digested HAMS was air-dried and sterilized by UV for 1 h.

The *in vitro* fermentation protocol was developed based on a previously described method (Wang et al., 2018) with modifications. In brief, a basal medium (pH 6.8) that contained tryptone (2.5 g/L), yeast extract (0.5 g/L) and mineral salts adapted from McSweeney et al. (2005) was used as fermentation medium. Resazurin solution was used as the oxidation-reduction indicator. The medium was boiled and cooled in the anaerobic hood, prior to the addition of cysteine HCl. Ingredients of the fermentation medium is included in Supplementary Table 1. Ten milliliter of medium was transferred under anaerobic conditions to Hungate tubes, which were stoppered and autoclaved.

Fecal sample and pre-digested HAMS were dissolved in anaerobic diluent (pH 6.8) for preparing the slurry. One mL of fecal slurry (with 100 mg of feces) and 0.5 ml of HAMS slurry (with 100 mg of HAMS) were injected to the fermentation tubes containing 10 ml of basal medium under anaerobic conditions. The level of HAMS used was selected to provide continuity with previous *in vitro* investigation in other subject populations (Edwards et al., 1996) and is not necessarily a concentration that would be achieved *in vivo*. Control fermentation tubes were prepared with 0.5 ml of anaerobic diluent in place of HAMS slurry. Following inoculation, Hungate tubes were incubated at 37°C for 24 h with constant shaking at 150 rpm.

### Determination of pH and SCFA Levels

Post-fermentation, samples were centrifuged at 13,000 *g*, 4°C, for 10 min. Supernatant pH was determined before and after fermentation using a pH meter (Mettler-Toledo Ltd.). Samples for SCFA measurement were prepared from pre- and post-fermentation supernatant using a water-extraction method modified from Zhao et al. (2006). 4-Methylvaleric acid (Sigma-Aldrich) was used as an internal standard. SCFA concentrations were determined by gas chromatography (GC, Agilent Technologies 7890A, Agilent Technologies, Santa Clara, CA, United States) fitted with a flame ionization detector (Agilent Technologies, Santa Clara, CA, United States). Details of GC column and running conditions were included in the method section of Supplementary Material.

### DNA Extraction

DNA extraction was performed using a Mo Bio PowerLyzer PowerSoil^®^ 96 Well DNA isolation kit (Mo Bio Laboratories). Manufacturer’s instructions were modified as follows: Samples were added into bead tubes with solution C1 and heated at 65°C for 10 min, prior to two cycles of bead beating at 6.5 m/s for 1 min using a FastPrep-24 bead beater (MP Biomedicals). DNA concentrations were quantified fluorometrically with a Quant-iT dsDNA Assay kit (Life Technologies).

### Quantitative PCR and 16S rRNA Gene Amplicon Sequencing

qPCR was performed to determine the absolute levels of total bacteria using methods described previously (Nadkarni et al., 2002). Amplicon sequencing of the V4 hypervariable region of the bacterial 16S rRNA gene was performed as described previously (Choo et al., 2015) using an Illumina MiSeq platform. Paired-end16S rRNA gene amplicon sequence reads were analyzed with quantitative insights into microbial ecology (QIIME) software (v1.8.0) (Caporaso et al., 2010). The datasets of 16S rRNA gene sequence for this study can be found in the NCBI Sequence Read Archive under accession number PRJNA516214.

### Statistical Analysis

Normality of data distribution was assessed using the Shapiro–Wilk test. Intergroup differences in bacterial load at baseline were assessed using Mann–Whitney *U*-test (pre-weaning vs. weaning) and in measures of fermentation outcomes using Wilcoxon rank-sum tests (HAMS vs. Control) (GraphPad Prism version 7.00). Intergroup differences in microbiota β-diversity were assessed using the permutational analysis of variance (PERMANOVA) model, based on the parameters’ permutation of residuals under a reduced model and a type III sum of squares (Primer-E v.7; Primer-E Ltd., Plymouth, United Kingdom) (Anderson and Walsh, 2013). Nonmetric multidimensional scaling (nMDS) plots were generated using weighted UniFrac distances. Differences of microbiota membership between pre-weaning and weaning groups at different taxonomic levels were analyzed by LEfSe (Galaxy application tool^1^) using linear discriminant analysis (LDA) scores > 2. Correlation between SCFA concentrations and taxa were performed using Spearman rank correlation (GraphPad Prism). Values in text were presented as median (interquartile range).

## Results

### Baseline Microbiota Composition

Thirty-four samples (11 pre-weaning, 23 weaning) that had sufficient volume for performing the *in vitro* fermentation were used in the study. Infant characteristics are provided in Table 1. Analysis was performed on all samples prior to, and following, *in vitro* fermentation. One pre-fermentation sample (weaning) failed to achieve sufficient sequence read depth and was excluded from further analysis. Analysis of the composition of fecal microbiota from Malawian infants (pre-fermentation) showed it to be dominated by members of the *Bifidobacterium, Veillonella*, and *Escherichia-Shigella* genera (data not shown).

**Table 1:** Participant characteristics.^1^

	Pre-weaning (*n* = 11)	Weaning (*n* = 23)
Age at sampling (month)	2.9 (1.5–4.5)	5.6 (3.0–10.5)
Weight (kg)	6.4 (4.5–7.5)	7.3 (5.0–10.3)
Length (cm)	61.5 (54.0–65.8)	67.0 (55.2–79.0)
Stunted^2^ (n)	2	1
Feeding practice	Exclusive breastfeeding	Mixed feeding of breast milk and complementary foods^3^


*^1^All infants were vaginally delivered and had no antibiotic intake for two weeks prior to sample collection (inclusion criteria). Data are presented as median (min–max). ^2^Stunting is defined as length-for-age below -2 z-scores of WHO Child Growth Standards. ^3^Main type of complementary foods include Cerelac (Nestle), Likuni Phala (Malawi product, Maize-based infant cereal) or home-made maize porridge.*

**FIGURE 1:**
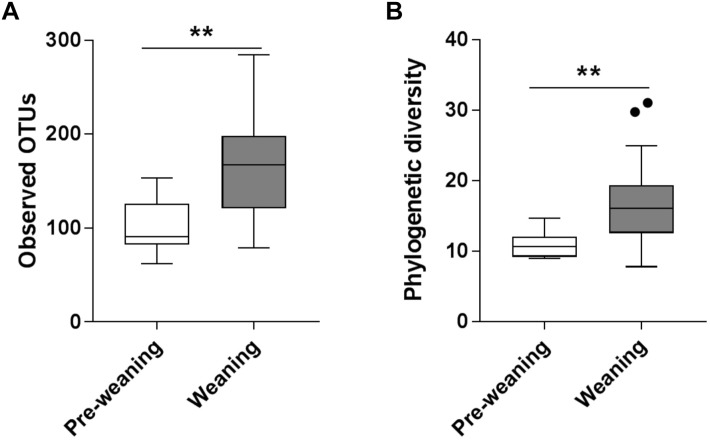
Alpha diversity of pre-weaning and weaning pre-fermentation fecal microbiota. **(A)** Taxa richness (observed OTUs) and **(B)** Faith’s phylogenetic diversity. Significance was determined by Mann–Whitney *U* test with *P* < 0.05. ^∗∗^*P* < 0.01.

**FIGURE 2:**
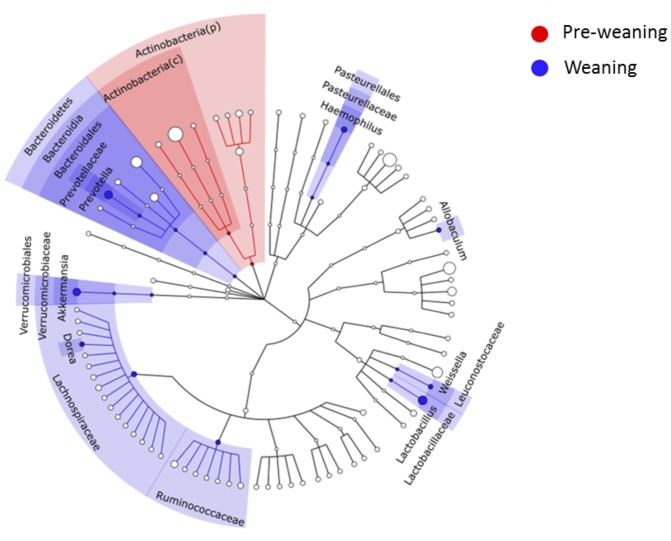
LEfSe cladogram showing microbial members at different taxonomical levels that differed between pre-weaning and weaning infants at baseline. A linear discriminant analysis score (LDA) cut-off of 2 was employed. c, class; p, phylum.

Substantial differences in fecal microbiota were observed between pre-weaning and weaning groups prior to fermentation. Weaning samples showed higher OTU richness (*P* = 0.0007, Figure 1A) and higher phylogenetic diversity compared to pre-weaning samples (*P* = 0.0006, Figure 1B). No significant difference was detected in the composition of the fecal microbiota as a whole in pre-weaning and weaning samples (based on weighted UniFrac distances) (PERMANOVA, *P* = 0.18, data not shown). However, significant differences in the relative abundance of specific taxa were identified (Figure 2). Samples from pre-weaning infants had a significantly greater relative abundance of the Actinobacteria phylum (LDA = 4.9, Figure 2), which were predominantly members of the *Bifidobacterium* genus. Weaning samples exhibited higher levels of *Prevotella, Lactobacillus, Allobaculum, Weissella, Dorea, Akkermansia*, and *Haemophilus* genera (LDA > 2, Figure 2). Most of these taxa were absent or present at extremely low relative abundance in pre-weaning samples, with the exception of *Lactobacillus*.

### Changes in pH and Bacterial Load Resulting From HAMS Fermentation

**FIGURE 3:**
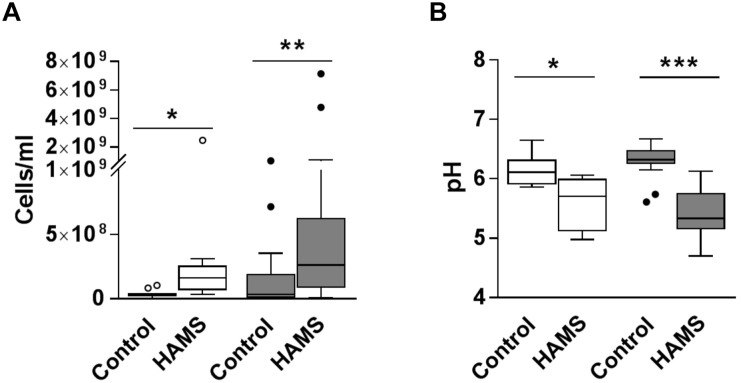
Total bacterial load and pH post-fermentation for pre-weaning (blank) and weaning (shaded) groups. **(A)** Total bacterial load post-fermentation for fecal samples fermented with and without HAMS (control). **(B)** pH post-fermentation for fecal samples fermented with HAMS and control. Significance was determined using Wilcoxon rank-sum tests with *P* < 0.05. ^∗^*P* < 0.05; ^∗∗^*P* < 0.01, ^∗∗∗^*P* < 0.001.

Fermentation of HAMS resulted in increased total bacterial load and reduced medium pH for both pre-weaning and weaning samples (Figure 3). Median total bacterial load increased 4.5-fold for pre-weaning (*P* = 0.002)and more than 7-fold for weaning (*P* = 0.001), compared to controls (Figure 3A). Correspondingly, pH of the fermentation medium dropped by 0.65 for pre-weaning (*P* = 0.001) and 0.99 units for weaning samples (*P* < 0.0001, Figure 3B). The change in bacterial load and pH were not significantly greater in the weaning group compared to the pre-weaning group.

### Changes in Fecal Microbiota Resulting From HAMS Fermentation

**FIGURE 4:**
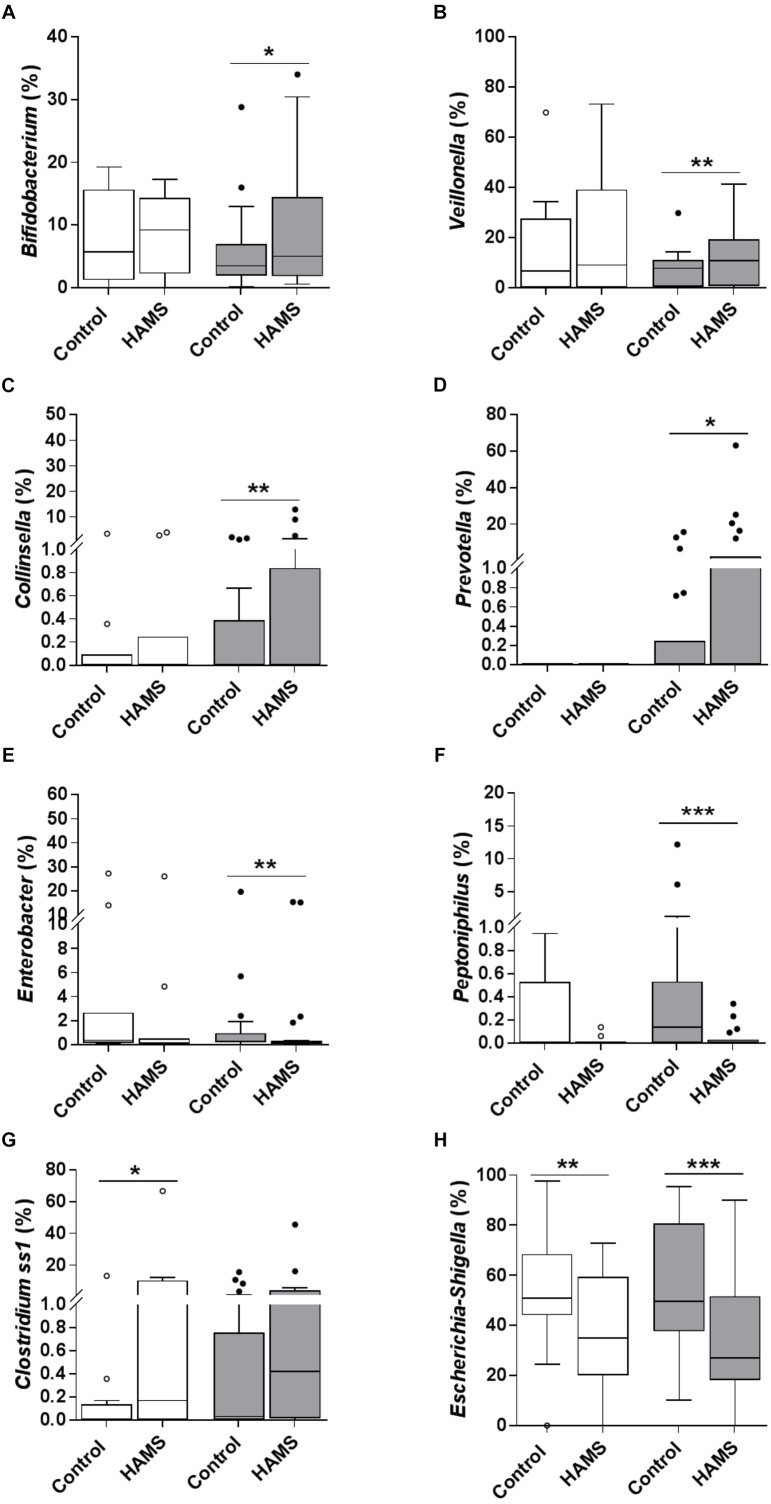
Taxa changed during fermentation with and without HAMS (control) for pre-weaning (blank) and weaning (shaded). Significance was determined using Wilcoxon rank-sum test with *P* < 0.05. ^∗^*P* < 0.05; ^∗∗^*P* < 0.01, ^∗∗∗^*P* < 0.001. **(A)** *Bifidobacterium*, **(B)** *Veillonella*, **(C)** *Collinsella*, **(D)** *Prevotella*, **(E)** *Enterobacter*, **(F)** *Peptoniphilus*, **(G)** *Clostridium* ss1, and **(H)** *Escherichia-Shigella*.

Fermentation of HAMS resulted in a significant shift in microbiota composition in weaning samples (weighted UniFrac Distance, HAMS vs. Control, PERMANOVA, *P* < 0.05, Supplementary Figure 1B). Changes in microbiota composition in pre-weaning samples did not achieve statistical significance (Supplementary Figure 1A). Specifically, the relative abundance of *Bifidobacterium, Veillonella, Collinsella*, and *Prevotella* were significantly higher in weaning samples compared to control following HAMS fermentation (Figure 4A–D), while *Enterobacter* and *Peptoniphilus* were significantly lower (Figure 4E,F). However, these taxa showed no significant differences in relative abundance compared to controls for the pre-weaning group (Figure 4A–F). In contrast, the relative abundance of *Clostridium* sensu stricto 1 (*Clostridium* ss1) increased significantly in pre-weaning group compared to control, but not in the weaning group (Figure 4G). Relative abundance of *Escherichia-Shigella* decreased in both pre-weaning and weaning samples compared to control (Figure 4H).

### Changes in SCFAs Resulting From HAMS Fermentation

**FIGURE 5:**
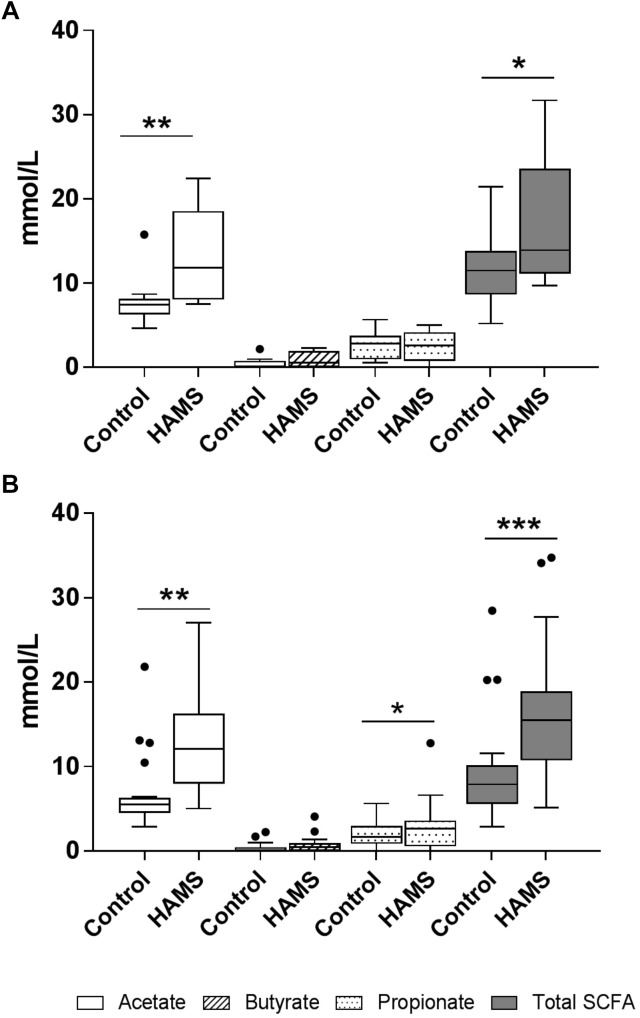
SCFA concentrations for pre-weaning **(A)** and weaning **(B)** groups post-fermentation with and without HAMS (control). Significance was determined using Wilcoxon rank-sum tests with *P* < 0.05. ^∗^*P* < 0.05; ^∗∗^*P* < 0.01, ^∗∗∗^*P* < 0.001.

HAMS fermentation resulted in significant increases in the levels of acetate compared to controls for both pre-weaning and weaning groups (Figure 5). Propionate concentrations increased in response to HAMS in the weaning group (Figure 5B), but not in the pre-weaning group (*P* = 0.70, Figure 5A). Total SCFA levels also increased in both pre-weaning and weaning groups (*P* = 0.0001, Figure 5A,B) largely reflecting increases in acetate. Butyrate levels did not increase significantly in either pre-weaning or weaning samples.

To identify the bacteria taxa that are likely to contribute to SCFA production, the relative abundance of taxa that showed a significant increase or decrease during HAMS fermentation was correlated to post-fermentation levels of acetate and propionate. Significant correlations were observed only for weaning samples. The relative abundances of *Veillonella* and *Prevotella* were positively correlated with levels of both acetate and propionate (Table 2). *Collinsella*, a genus belonging to the Actinobacteria phylum, also showed a positive correlation with propionate (Table 2). *Escherichia-Shigella* and *Clostridium* ss1, which showed significant decreases in relative abundance during HAMS fermentation, were negatively correlated with post-fermentation propionate concentration (Table 2).

**Table 2:** Correlation between SCFAs and taxa that were changed by HAMS during fermentation.^1^

SCFA	Taxon	*r*_s_	*P*-value
Acetate			
	*Prevotella*	0.42	0.044
	*Veillonella*	0.50	0.016
	*Clostridium ss1*	-0.46	0.028
Propionate			
	*Collinsella*	0.55	0.0065
	*Prevotella*	0.45	0.032
	*Veillonella*	0.78	<0.0001
	*Escherichia-Shigella*	-0.55	0.0066


*^1^Only significant correlations from weaning group are shown. No significant correction was observed in pre-weaning group using Spearman rank correlation test. Clostridium ss1, Clostridium sensu stricto1.*

## Discussion

We assessed the capacity of the fecal microbiota from infants in a rural location in Malawi to ferment the type 2 RS, HAMS. The study population was subject to many of the factors that contribute to the burden of disease from EED in the developing world, including limited sanitation, water treatment processing, and public health resources (Owino et al., 2016).

Overall, the characteristics of the fecal microbiota of these Malawian infants were in keeping with those reported for infants in developed countries (Backhed et al., 2015; Murphy et al., 2017). Consistent with previous analyses of infant fecal microbiota composition (Backhed et al., 2015), pre-weaning samples exhibited significantly lower microbiota richness and diversity, compared to samples from weaning infants. The microbiota of pre-weaning fecal samples were dominated by *Bifidobacterium* and *Veillonella*, typical early colonizers of the human gut (Murphy et al., 2017). While many members of the *Bifidobacterium* genus are able to produce acetate through the degradation of a wide range of carbohydrates, including starch (Pokusaeva et al., 2011), particular species that are common in the infant gut (e.g., *Bifidobacterium longum*) may lack this capacity (Ryan et al., 2006), instead primarily metabolizing human milk oligosaccharides (Garrido et al., 2011; Ruiz-Moyano et al., 2013).

*Veillonella* species, in contrast, are unable to utilize carbohydrates, and instead exploit lactate and succinate produced by other taxa during carbohydrate-degradation (Rainey, 2009). Common starch-utilizing taxa, including the *Prevotella* and *Dorea* genera, were not detected in the pre-weaning samples.

The fecal microbiota of weaning infants, while still dominated by the *Bifidobacterium* and *Veillonella* genera, contained an abundance of carbohydrate-degrading bacteria. These included the *Prevotella, Bacteroides*, and *Faecalibacterium* genera. The difference in composition between the infant groups is likely to reflect the introduction of solid foods during weaning, a process that helps to shape the developing gut microbiota by providing new substrates for bacterial growth (Fallani et al., 2011).

An *in vitro* fermentation model that mimics fermentation in the infant gut allows the preliminary assessment of the microbial capacity to utilize HAMS in a safe and defined pre-clinical setting. Compositional differences between pre-weaning and weaning fecal microbiota were reflected in their response to HAMS during *in vitro* fermentation. HAMS fermentation occurred in both sample groups, as indicated by an increase in bacterial cell numbers, a drop in pH, and an increase in SCFA concentrations. However, fecal microbiota β-diversity (an indicator of change in overall microbiota composition between pre- and post-fermentation) with HAMS were modest and significant only in the weaning group, suggesting that HAMS does not represent a strong selective pressure across all taxa, with an effect that is limited to a discrete group of taxa. While post-fermentation concentrations of acetate were increased in both groups, the correlations with bacterial taxa were substantially weaker for pre-weaning microbiota, an observation that might be explained by the limited presence of acetate-producers in the immature pre-weaning microbiota. For example, the absence of the acetate-producing *Prevotella* genus in breast-fed pre-weaning samples might be explained by its association with plant-derived dietary carbohydrates (Martinez et al., 2015; De Filippis et al., 2016). Similarly, the introduction of complementary foods is likely to underpin the increased diversity of fermentative taxa in the weaning group more widely.

Propionate production was only observed in weaning samples, and was positively correlated with *Prevotella, Collinsella*, and *Veillonella* relative abundance. The apparent involvement of these three taxa in SCFA production might reflect a metabolic interaction between members of these genera. Many *Collinsella* and *Prevotella* species do not produce propionate, but can produce substantial amounts of succinate through carbohydrate fermentation (Kageyama and Benno, 2012; De Vadder et al., 2016). In contrast, most of the members of the *Veillonella* genus are unable to ferment carbohydrates, but can produce propionate through the fermentation of succinate (Rainey, 2009).

The absence of significant butyrate biosynthesis in either pre-weaning or weaning samples during HAMS fermentation, with SCFA production largely limited to acetate and propionate, is consistent with previous infant studies (Parrett et al., 2003; Scheiwiller et al., 2006) and is in contrast to adults (Parrett and Edwards, 1997). Indeed, bacterial taxa that are responsible for butyrogenesis in the human gut, including *Faecalibacterium prausnitzii, Eubacterium rectale, Roseburia faecis*, and other members of *Clostridium* clusters IV, XIVa, and XVI (Louis et al., 2004; Louis et al., 2010), were absent or present in very low relative abundances in the infant fecal samples assessed here.

*Bifidobacterium* is typically the most prevalent genus in the infant gut microbiota (Kleessen et al., 1995) and can provide protection against infection by enteropathogens through the production of acetate, and the promotion of gut barrier function (Fukuda et al., 2011). Resistant starch has been shown to increase the relative abundance of bifidobacteria in a porcine model (Fouhse et al., 2015). In our study, we observed a substantial increase in bifidobacteria in the weaning group only, but the absence of such an effect in pre-weaning infants. This difference is likely to reflect the inability of bifidobacteria to utilize HAMS, and their reliance on the presence of primary polysaccharide degraders (Liu et al., 2015); which were only observed following the introduction of complementary foods.

The observed decrease in the relative abundance of *Escherichia-Shigella* resulting from HAMS fermentation in both pre-weaning and weaning groups might be of clinical relevance. The *Escherichia-Shigella* group contains many pathogens that are strongly associated with intestinal infection and EED risk, including pathogenic *Escherichia coli* (*E. coli*) (Crane et al., 2015). A significant negative correlation was observed between *Escherichia-Shigella* relative abundance and the propionate biosynthesis in the weaning group, and a similar relationship with acetate levels trended toward significance (data not shown). This effect might reflect the production of acid during fermentation (including lactate and SCFAs) and the resulting decrease in pH. It has been shown *in vitro* that the growth of pathogenic *E. coli* can be substantially suppressed by low pH and presence of SCFAs (Shin et al., 2002). Additionally, the ability of high SCFA levels and low pH to inhibit intestinal colonization by pathogenic *Shigella sonnei* and *E. coli* has been demonstrated previously in a mouse model (Pongpech and Hentges, 1989). The ability of HAMS fermentation to inhibit the growth of *Escherichia-Shigella* might therefore provide additional clinical benefit.

Our findings suggest that HAMS supplementation in Malawian infants might provide benefits to gut health, and by extension, early life development. Whilst ensuring that exclusive breastfeeding for the first 6 months of life is not disturbed, there is already strong support for the inclusion of RS as HAMS into ORS (Ramakrishna et al., 2008; Binder et al., 2014). The consumption of ORS to prevent dehydration when diarrhea is present is recommended in the WHO guidelines for exclusive breastfeeding (Victora et al., 2016). The ability of SCFAs to stimulate colonic sodium and fluid absorption via acyclic AMP-independent mechanism (Binder et al., 2014) warrants further consideration in support of including HAMS in the ORS formulation. The potential microbiome-mediated benefits of such an intervention should also be considered further. In weaning and older infants, replication of our microbiological findings through feeding studies in Malawian infants, in conjunction with the demonstration of associated health benefits would support the inclusion of HAMS as a supplement to the typical diet for this population.

This study had a number of limitations that should be considered. First, the weaning group contained infants transitioning to a range of foods, and with likely variation in the relative consumption of breast milk. These are factors that are likely to influence microbiota composition. However, the cohort was not sufficiently large, nor was food intake data sufficiently detailed, to relate dietary habit to HAMS response within this population. Second, while considerable efforts were made to ensure the integrity of samples shipped from Malawi to Australia for analysis, it is not possible to determine with certainty whether this process effected overall microbial viability or the relative viability of specific taxa. Third, the *in vitro* fermentation system used to assess HAMS utilization does not fully replicate the conditions within the infant gut. This approach merely provides a broad indication of the capacity of gut microbiota to utilize HAMS to produce SCFA, and the taxa that are largely responsible. Fourth, HAMS was assessed relative to a no substrate control, rather than alternative fermentation substrates. Fifth, the extent to which HAMS supplementation results in increased SCFA production *in vivo* can only be determined through an infant feeding study. Such a study would further allow response to HAMS supplementation to be linked to potential enteric health and developmental benefits.

In summary, the fecal microbiota of Malawian infants before and during weaning have the ability to ferment HAMS and produce beneficial metabolites, in particularly acetate. The capability of propionate production through HAMS fermentation may only develop when bacterial species that represent primary starch degraders begin to colonize the gut following the introduction of complementary foods. Consistently, the impact of HAMS on the composition of the fecal microbiota was more profound during weaning. In particular, HAMS was associated with the promotion of important commensal bacteria, including members of the *Bifidobacterium, Prevotella, Veillonella, Collinsella*, and *Clostridiums ss1* genera, and a reduced prevalence of *Escherichia-Shigella, Enterobacter*, and *Peptoniphilus*. These findings support the concept that HAMS might be an effective and beneficial fermentable polysaccharides for feeding to infants during weaning. *In vivo* studies to explore this potential are therefore indicated.

## Data Availability

The datasets generated for this study can be found in NCBI Sequence Read Archive, PRJNA516214.

## Ethics Statement

This study was conducted according to the principles expressed in the Declaration of Helsinki, and all research procedures were approved by the Southern Adelaide Clinical Human Research Ethics Committee (SACHREC) for Flinders University (504.14-HREC/14/SAC/541) and the University of Malawi, College of Medicine Research Ethics Committee (COMREC) (P.08/15/1781). In addition, a study approval letter was provided through the Neno District Health Office, authorizing the involvement of the Neno District Hospital. Written informed consent was obtained from all caregivers of the participating children.

## Author Contributions

GY, EM, GG, CC, and KK designed the study. EM, KK, and NK collected the samples. YW, LL, AR, and AS conducted the related wet-lab work. YW, GR, and GA analyzed the data. YW, GR, EM, and KK drafted the manuscript, which was received and approved by all authors.

## Conflict of Interest Statement

The authors declare that the research was conducted in the absence of any commercial or financial relationships that could be construed as a potential conflict of interest.

## Footnotes

1 http://huttenhower.sph.harvard.edu/galaxy/
