# Supplementary material for: The Capacity of the Fecal Microbiota From Malawian Infants to Ferment Resistant Starch
Source: Front Microbiol. 2019 Jun 26;10:1459. doi: 10.3389/fmicb.2019.01459 (PMC6611432; doi:10.3389/fmicb.2019.01459)
Supplement: Supplementary file 1 [file Data_Sheet_1.docx]

**Supplementary Material**

**The capacity of the faecal microbiota from Malawian infants to ferment resistant starch**

Yanan Wang, Elissa K. Mortimer, Kondwani G.H. Katundu, Noel Kalanga, Lex E.X. Leong, Geetha L. Gopalsamy, Claus T. Christophersen, Alyson C. Richard, Aravind Shivasami, Guy C.J. Abell, Graeme P. Young, Geraint B. Rogers

**Supplementary Methods**

***Gas chromatography method for SCFA determination:***

Gas chromatography (GC) was performed on an Agilent Technologies 7890A. The gas chromatograph fitted with a flame ionization detector and 7683B autosampler (Santa Clara, CA, USA). The capillary column used was a SGE Analytical BP21, (15 m x 0.25 mm i.d.) with a 0.25 µm film thickness

The samples were analysed as supplied. Split injection was used with a split ratio of 50:1.

Injection port temperature of 250 ^o^C. The carrier gas was Helium in constant flow mode at 1 mL/min. The injection Volume was1 µL. The GC temperature program was: Initial temperature: 100 ^o^C for 6 min.; ramp: 30 ^o^C/min; final temperature: 220 ^o^C for 2 min. FID Temperature: 280 ^o^C; H_2_ Flow: 40 mL/min; Air Flow: 450 mL/min; Makeup gas (N_2_): 50 mL/min

***Pre-digestion procedure***

HAMS (Hylon VII, Ingredion Incorporated, Westchestor, IL, USA) used in the *in vitro* fermentation was pre-digested with an *in vitro* method modified from Woolnough et al. ^(1)^ simulating gastric and small intestinal starch digestion in infants. In brief, 2.5 g of HAMS was incubated with 12.5 ml of pepsin solution (1 mg/ml in 0.02 M HCl) at 37°C for 30 min with shaking (150 rpm). After the incubation, pH was adjusted to 6.0 with the addition of 12.5 ml of 0.02M sodium hydroxide and 62.5 ml of 0.2 M of sodium acetate, followed by the addition of 12.5 ml of enzyme mixture containing 1.5 U/ml α-amylase (Sigma-Aldrich, St. Louis, MO, USA) and 200 U/ml of amyloglucosidase (Sigma-Aldrich, St. Louis, MO, USA). The mixture of samples and digestive enzymes was incubated at 37°C with shaking (150 rpm) for 5 h. Subsequently, samples were precipitated overnight with 400 ml of ethanol (100%). The precipitates were collected and washed three times with 100 ml of 80% ethanol and one time with 25 ml of sodium acetate by centrifugation. Pellets recovered from centrifugation were air-dried, sterilized by UV for 1 h and stored at room temperature before use.

**Supplementary Table 1.** Ingredients of basal medium and anaerobic diluent used in the *in vitro* fermentation. Ingredients are per litre of distilled water.

|  | **Medium** | **Anaerobic diluent** |
| --- | --- | --- |
| Tryptone | 2.5g | - |
| Yeast extract | 0.5g | - |
| K_2_HPO_4_ | 1.20g | 0.46g |
| NaCl | 1.21g | 0.46g |
| (NH_4_)_2_SO_4_ | 0.60g | 0.23g |
| MgSO_4_.7H2O | 0.28g | 0.095g |
| CaCl_2_.2H2O | 0.16g | 0.061g |
| N(CH₂CO₂H)₃ | 1.5mg | - |
| MnSO_4_.7H2O | 0.5mg | - |
| FeSO_4_.7H2O | 0.1mg | - |
| ZnSO_4_.7H_2_O | 0.1mg | - |
| CoCl_2_.6H_2_O | 0.1mg | - |
| NiSO_4_.6H_2_O | 0.03mg | - |
| Na_2_SeO_3_ | 0.02mg | - |
| AlK(SO_4_)_2_.12H_2_O | 0.01mg | - |
| H_3_BO_3_ | 0.01mg | - |
| CuSO_4_.5H2O | 0.01mg | - |
| Haemin | 0.05g | - |
| Resazurin | 0.1mg | 0.1mg |
| L-cysteine HCl | 0.25g | 0.25g |

**
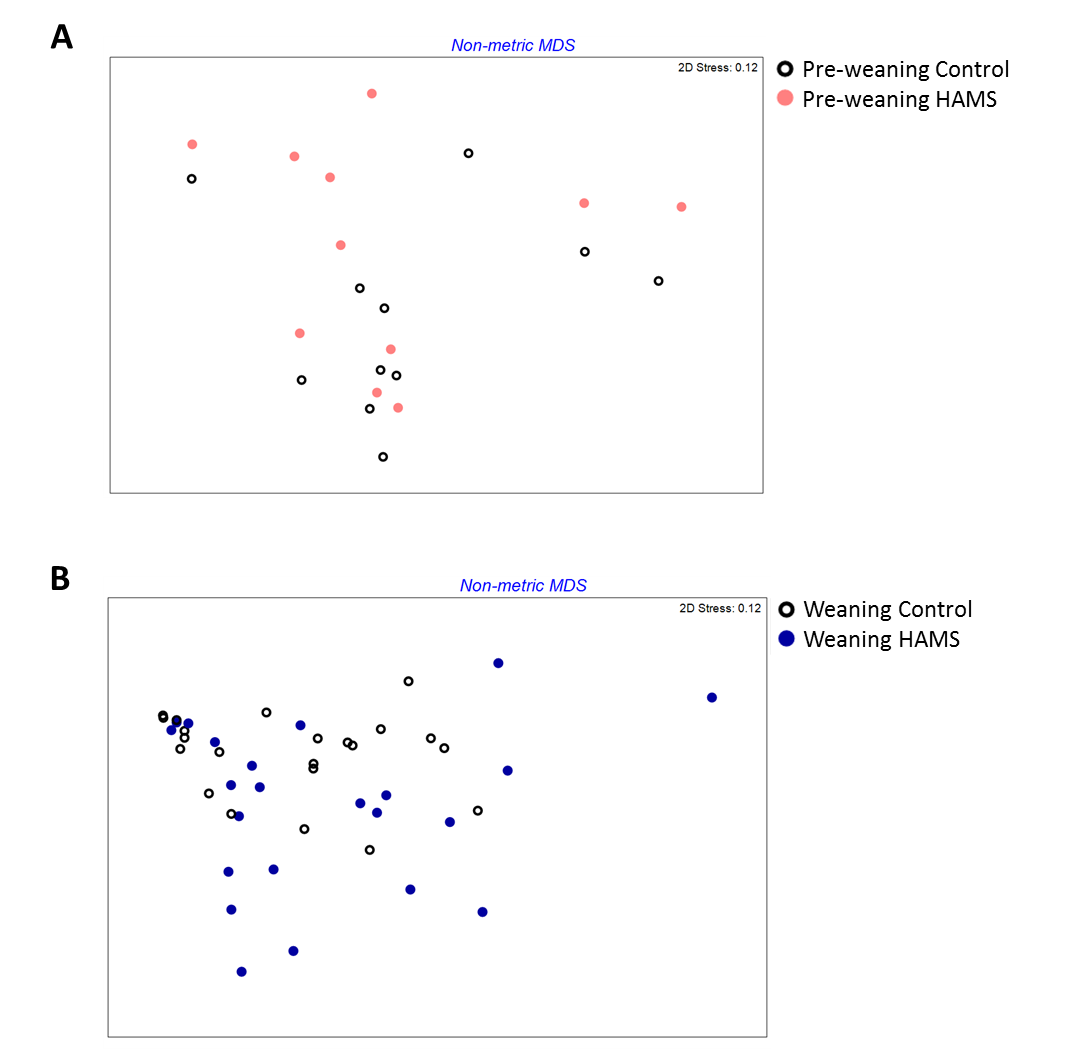
**

**Supplementary Figure 1.** nMDS plot derived from weighted UniFrac distance showing differences of microbiota with HAMS or without HAMS (control) in pre-weaning (A)and weaning (B) groups post-fermentation.

**References for Supplemental Materils**

1. Woolnough JW, Bird AR, Monro JA *et al.* (2010) The effect of a brief salivary alpha-amylase exposure during chewing on subsequent in vitro starch digestion curve profiles. *Int J Mol Sci* **11**, 2780-2790.
